# Reducing fall injuries with better data

**DOI:** 10.1186/s40621-023-00481-2

**Published:** 2023-12-21

**Authors:** David Hemenway, Elizabeth W. Peterson, Jonathan Howland

**Affiliations:** 1 Department of Health Policy and Management, Harvard T.H. Chan School of Public Health, 677 Huntington Avenue, Boston, MA 02115 USA; 2 Department of Occupational Therapy, University of Illinois at Chicago, Chicago, IL USA; 3 Department of Emergency Medicine, Boston University Chobanian and Avedisian School of Medicine, Boston, MA USA

**Keywords:** Falls, Fall deaths, Fall injuries, Surveillance systems, Data systems, Standards, Certification

## Abstract

**Background:**

Fall deaths in the USA almost tripled in the twenty-first century. While various interventions have been effective in reducing fall deaths, they have failed to make a substantial impact at a population level.

**Main body:**

An overarching factor that has been relatively neglected in fall injury prevention is the need for more and better data. We need better data on the causes and circumstances of older adult fall deaths. While there are excellent national surveillance systems on the circumstances of other injury deaths (e.g., motor vehicle crashes, suicides, and homicides), such a system is lacking for fall deaths. These other data systems have been instrumental in indicating and evaluating policies that will reduce injury. It is also important to provide consumers with better information concerning the many products that affect the likelihood of fall injury (e.g., flooring, hip protectors, footwear). Automotive buyers are provided with relevant up-to-date make-model safety information from crash tests and real-world performance. Such information not only helps protect buyers from purchasing dangerous products, but it provides producers with the incentive to make ever safer products over time.

**Conclusion:**

We believe that creation of a national surveillance system on the circumstances of fall deaths, and increased testing/certifying of fall-related products, are two steps that would help create the conditions for continuous reductions in fall fatalities. Fall prevention should apply some of the same basic strategies that have proved effective in addressing other injuries.

## Background

In the past two decades (2001–2021), the number of Americans dying from unintentional falls increased from 15 thousand to over 44 thousand as the crude death rate rose from 5.3 (per 100,000 population) to 13.5 (Centers for Disease Control and Prevention 2023). Deaths from falls are expected to continue to rise as the baby boom generation moves through retirement age and experiences the frailty, chronic conditions, and medications that are associated with increased fall risk.

While various interventions have been effective in reducing fall deaths (Montero-Odasso et al. 2022)—by reducing intrinsic risk factors (e.g., improving strength, vision, balance) and/or extrinsic risk factors (e.g., shaping the built environment so falls are less likely and less serious)—they have failed to make a substantial impact at a population level. In this essay, we focus on an overarching factor we believe has been relatively neglected but is crucial for reducing fall fatalities: data. We need better data both on (a) the causes and circumstances of fall deaths and on (b) the types of products that can reduce fall injuries.

## Main text

### National fall fatality surveillance system

The first step in the public health approach to fall injury prevention is to create a national surveillance system to provide information on the circumstances of fall fatalities. The existing WISQARS™ injury surveillance system provides demographic information (e.g., age, sex, region) on fatal fall victims but does not yield the circumstantial data needed to inform high-impact interventions. By contrast, motor vehicle fatalities have such a data system (the national Fatality Analysis Reporting System or FARS) as do suicides and homicides (the National Violent Death Reporting System, or NVDRS).

Federal data systems on the circumstances of fatalities have been instrumental in indicating and evaluating policies intended to reduce injuries. For example, data from FARS showed that 16-year-old drivers were at high risk of death and were at the highest risk in two situations—when they were driving at night and when only other teenagers were in the vehicle. With that information, Michigan initiated a graduated driver licensing program in which 16-year-olds are allowed to drive, but not in those two situations. Analyses using FARS data found that the program reduced fatalities among these drivers by 30%. Other states quickly adopted similar measures, with similar success, and soon all 50 states had graduated driver licensing programs, saving many lives (Hemenway 2009).

We need a similar national data system for fall deaths that will provide consistent and comparable circumstantial information across states and over time. The method by which the NVDRS was created might provide a template for creating such a system. Calls for a firearm data system (Teret et al. 1992) led a consortium of foundations to provide funding for three inter-related private activities: creating a pilot for the program across a variety of states, creating descriptive materials (e.g., pamphlets, quizzes) to demonstrate the social benefits of the system, and organizing lobbying support from scores of key private associations (e.g., American Medical Association, American Bar Association).

With funding, an important early step was a national conference that brought together experts in injury surveillance, research, and policy from government, academia, and other non-profits. At this conference, issues were resolved, barriers overcome, and key strategic decisions agreed upon—such as to focus on fatalities only, to include non-firearm violent deaths in the system, and that the Centers for Disease Control and Prevention (CDC) was the appropriate federal agency for the system. Within 3 years, the nascent NVDRS was housed at the CDC and was beginning to collect state-level data (Hemenway et al. 2009).

The new fatality system for fall deaths, like NVDRS, could be housed at the CDC, or in an independent agency (as FARS is). The cost would be but a rounding error in the federal budget; the FY 2023 budget for NVDRS was $24.5 million.

A national data system on the circumstances of fall deaths can increase the consistency of coroner/medical examiner reports (Walsh et al. 2007) and improve on the accuracy of Vital Statistics. NVDRS helped demonstrate that death certificate data alone (WISQARS) resulted in a large undercounting of both unintentional firearm fatalities to children and victims killed by law enforcement officers. NVDRS is now the accurate federal source for such data. Similarly, a falls fatality data system may improve the accuracy of vital statistics data–including the data reported in the first sentence of this Commentary.

### Consumer information

For markets to work well, consumers need good information. Good product quality information can protect buyers from unsafe products and provide suppliers with incentives to make ever safer products over time. Accurate information on fall causation could stimulate the development of new products to prevent or mitigate falls and accelerate the deployment of existing fall prevention/mitigation products.

The twenty-first-century reduction in motor vehicle deaths shows that it is possible to create an environment that creates incentives for continuous reductions in injury deaths (Hemenway and Lee 2022). The key to providing the right incentives appears to have been the combination of an excellent national data system (FARS) along with data on product safety that is continually provided to buyers.

The National Highway Traffic Safety Administration (NHTSA) (that has a mission to prevent traffic injuries) along with the Insurance Institute for Highway Safety (IIHS) (that crash-tests cars and rates vehicles), provide car buyers with relevant up-to-date make-model safety information from crash tests and real-world performance. Car manufacturers, that historically resisted competing on safety, now actively promote new safety features. Cars today are much safer than they were in 2000. For example, automatic emergency braking, blind spot detection, side airbags, and rear-facing cameras are now commonly available.

Many products potentially affect the likelihood of experiencing fall-related injuries including flooring, hip protectors, and footwear. It would be useful for older Americans to know what shoes are the most slip resistance and for suppliers and general contractors to have better information and stronger incentives to provide effective impact absorbing surfaces in high-risk spaces.

It would be ideal to have a NHTSA-type federal agency whose mission is to reduce fall injuries, along with an insurance-sponsored non-profit institute that evaluates the effectiveness of fall prevention initiatives and promotes policies that reduce the likelihood and severity of serious falls.

Standards and certification help ensure that safety products are effective. Many public and private organizations provide standards for industries, based on testing and other research. UL standards for electrical equipment are one example. The US federal government helped create voluntary quality standards for fire safe cigarettes. These standards were then mandated throughout the USA, reducing the likelihood of cigarette-caused fire deaths.

## Conclusion

The importance of systematically collected data on the circumstances of injury is well known. In its 1985 seminal report *Injury in America*, the National Research Council and the Institute of Medicine concluded that the “development of effective intervention strategies requires an adequate national surveillance system for monitoring injuries…” (Committee on Trauma Research, Commission on Life Sciences 1985) and the “lack of brand or even generic information on products associated with injuries is clearly a major barrier to the prevention of injuries” (Committee on Trauma Research, Commission on Life Sciences 1985). The fall prevention field has fallen behind in its ability to provide researchers and the marketplace with the types of information needed to counter current global trends in fall-related death. We should apply to falls the same strategies that have been effective in addressing other injuries.

## Data Availability

Not applicable.

## Abbreviations

CDC: Centers for Disease Control and Prevention
FARS: Fatality Analysis Reporting System
IIHS: Insurance Institute for Highway Safety
NHTSA: National Highway Traffic Safety Administration
NVDRS: National Violent Death Reporting System
WISQARS™: Web-based Injury Statistics Query and Reporting System

## References

[CR1] Centers for Disease Control and Prevention. WISQARS https://www.cdc.gov/injury/wisqars/fatal/index.html. Accessed June 2023.

[CR2] Committee on Trauma Research, Commission on Life Sciences (1985). National Research Council, & Institute of Medicine. Injury in America.

[CR3] Hemenway D (2009). While we were sleeping: success stories in injury and Violence prevention.

[CR4] Hemenway D, Barber CW, Gallagher SS, Azrael DR (2009). Creating a national violent death reporting system: a successful beginning. Am J Prev Med.

[CR5] Hemenway D, Lee LK (2022). Lesson from the continuing motor vehicle success. Inj Prev.

[CR6] Montero-Odasso M, van der Velde N, Martin FC, et al. Task force on global guidelines for falls in older adults. World guidelines for falls prevention and management for older adults: a global initiative. Age Aging 2022.

[CR7] Teret SP, Wintemute GJ, Beilenson PL (1992). The firarm fatality reporting system: a proposal. JAMA.

[CR8] Walsh S, Dignan M, Caldwell G (2007). The PAPM, diffusion theory, and violent death surveillance. Am J Health Behav.

